# Recovery from acute kidney injury as a potent predictor of survival and good neurological outcome at discharge after out-of-hospital cardiac arrest

**DOI:** 10.1186/s13054-019-2535-1

**Published:** 2019-07-15

**Authors:** Yoo Seok Park, Yoon Hee Choi, Je Hyeok Oh, In Soo Cho, Kyoung-Chul Cha, Byung-Sun Choi, Je Sung You

**Affiliations:** 1 Department of Emergency Medicine, Yonsei University College of Medicine, Seoul, Republic of Korea; 2 Department of Emergency Medicine, Ewha Womans University Medical Center and Ewha Womans University Mokdong Hospital, Seoul, Republic of Korea; 3 Department of Emergency Medicine, Chung-Ang University College of Medicine, Seoul, Republic of Korea; 4 Department of Emergency Medicine, Hanil General Hospital, Seoul, Republic of Korea; 5 Department of Emergency Medicine, Yonsei University Wonju College of Medicine, Wonju, Republic of Korea; 6 Department of Preventive Medicine, Chung-Ang University College of Medicine, Seoul, Republic of Korea

**Keywords:** Acute kidney injury, Out-of-hospital cardiac arrest, Targeted temperature management, Therapeutic hypothermia, Survival rate

## Abstract

**Background:**

Acute kidney injury (AKI) after out-of-hospital cardiac arrest (OHCA) is a well-known predictor for mortality. However, the natural course of AKI including recovery rate after OHCA is uncertain. This study investigated the clinical course of AKI after OHCA and determined whether recovery from AKI impacted the outcomes of OHCA.

**Methods:**

This retrospective multicentre cohort study included adult OHCA patients treated with targeted temperature management (TTM) between January 2016 and December 2017. AKI was diagnosed using the Kidney Disease: Improving Global Outcomes criteria. The primary outcome was the recovery rate after AKI and its association with survival and good neurological outcome at discharge.

**Results:**

A total of 3697 OHCA patients from six hospitals were screened and 275 were finally included. AKI developed in 175/275 (64%) patients and 69/175 (39%) patients recovered from AKI. In most cases, AKI developed within three days of return of spontaneous circulation [155/175 (89%), median time to AKI development 1 (1–2) day] and patients recovered within seven days of return of spontaneous circulation [59/69 (86%), median time to AKI recovery 3 (2–7) days]. Duration of AKI was significantly longer in the AKI non-recovery group than in the AKI recovery group [5 (2–9) vs. 1 (1–5) days; *P* < 0.001]. Most patients were diagnosed with AKI stage 1 initially [120/175 (69%)]. However, the number of stage 3 AKI patients increased from 30/175 (17%) to 77/175 (44%) after the initial diagnosis of AKI. The rate of survival discharge was significantly higher in the AKI recovery group than in the AKI non-recovery group [45/69 (65%) vs. 17/106 (16%); *P* < 0.001]. Recovery from AKI was a potent predictor of survival and good neurological outcome at discharge in the multivariate analysis (adjusted odds ratio, 8.308; 95% confidence interval, 3.120–22.123; *P* < 0.001 and adjusted odds ratio, 36.822; 95% confidence interval, 4.097–330.926; *P* = 0.001).

**Conclusions:**

In our cohort of adult OHCA patients treated with TTM (*n* = 275), the recovery rate from AKI after OHCA was 39%, and recovery from AKI was a potent predictor of survival and good neurological outcome at discharge.

**Electronic supplementary material:**

The online version of this article (10.1186/s13054-019-2535-1) contains supplementary material, which is available to authorized users.

## Background

Development of acute kidney injury (AKI) after out-of-hospital cardiac arrest (OHCA) is a well-known predictor for mortality and poor neurological outcomes in both adult and paediatric populations [1–6]. However, the natural course of AKI in terms of its recovery rate after OHCA is uncertain. Although the incidence and onset of AKI development have been reported several times, recovery rate after AKI has not been reported to date [3]. If we know the natural course of AKI after OHCA, it will be helpful in making appropriate clinical decisions such as determining the initiation time for renal replacement therapy (RRT) and making a prognosis in OHCA patients with AKI development. We hypothesised that recovery from AKI may affect the survival discharge and neurological status at discharge in OHCA patients treated with targeted temperature management (TTM). Therefore, this study investigated the clinical course of AKI (from development to recovery) after OHCA treated with TTM and determined whether recovery from AKI has an impact on the outcomes of OHCA after treatment with TTM.

## Methods

### Study design and setting

This was a retrospective, multicentre, cohort study. Data were collected by the review of medical record from the emergency departments of six academic hospitals located in South Korea. The study design and plan were approved by the institutional review boards of each hospital. Written informed consent was waived by the institutional review boards. We searched the medical records using the following diagnostic terminology: ‘death on arrival’, ‘cardiac arrest with successful resuscitation’, ‘cardiac arrest, unspecified’, ‘respiratory arrest’ and ‘post-resuscitation state’.

### Study population

All adult patients who visited the emergency departments of the six hospitals for OHCA between January 2016 and December 2017 and were treated with TTM were screened. The following patients were excluded: patients aged < 19 years; patients reported dead on arrival to the hospital and did not receive cardiopulmonary resuscitation (CPR); patients who did not achieve return of spontaneous circulation (ROSC) despite performing CPR; patients who did not receive TTM despite achieving ROSC; patients who were diagnosed with end-stage renal disease (ESRD) with dialysis (peritoneal dialysis or haemodialysis) before developing cardiac arrest; patients who had a do-not-attempt resuscitation (DNAR) order prior to the development of cardiac arrest; patients who had acute intracranial haemorrhage or acute ischemic stroke; and patients who had active bleeding.

### Definition of the development of and recovery of AKI

The definition and staging of AKI were based on the diagnostic criteria stipulated in the Kidney Disease: Improving Global Outcomes (KDIGO) guidelines (Additional file 1) [7]. Development of AKI was diagnosed if one of the criteria was fulfilled (serum creatinine or hourly urine output). If both criteria, the one for serum creatinine and hourly urine output, were fulfilled to diagnose different stages of AKI development, higher stage of AKI was diagnosed. If the data for the serum creatinine level within the last 3 months before the development of cardiac arrest were available, it was regarded as baseline serum creatinine. If this data were unavailable, the lower value between the estimated serum creatinine level and the first measured serum creatinine after achieving ROSC was regarded as baseline serum creatinine [7, 8]. Recovery from AKI was characterised by the fulfilment of all of the following conditions (absence of AKI criteria): serum creatinine decreased below the level determined in the definition of stage 1 AKI and hourly urine output was maintained over 0.5 mL/kg/h for ≥ 12 h [9].

### Variables

Primary outcome variables included the dates of AKI development and recovery, the duration of AKI, recovery rate, and survival discharge. If the AKI stage changed after the initial diagnosis, the highest stage of AKI was recorded. Secondary outcome variables included the following variables recommended by the Utstein resuscitation registry templates for OHCA: age (years), sex (male or female), weight (kg), past medical history (hypertension, diabetes mellitus, heart failure, or chronic kidney disease), witnessed arrest (witnessed or not witnessed), arrest location (home, workplace, sports or recreation, street or highway, public building, nursing home, and educational institution), bystander CPR (yes or no), first monitored rhythm (ventricular fibrillation, pulseless ventricular tachycardia, pulseless electrical activity, and asystole), cause of arrest (medical, trauma, drug overdose, drowning, electrocution and asphyxia), response time (time interval from call to visit), defibrillation time (time interval from call to the time the first shock), CPR duration (the sum of the prehospital and emergency room CPR times, min), epinephrine dose (mg), coronary angiography (yes or no), extracorporeal membrane oxygenation (yes or no), and targeted temperature of TTM (33 °C vs. 36 °C). The target temperature was determined by the physicians in charge of managing the patients at each institution because there is no difference in the outcomes between 33 °C and 36 °C. Therefore, a wide range of target temperatures [from 32 °C to 36 °C] are recommended in institutional standard operating procedures since the publication of the 2015 guidelines) [10, 11], maintenance period of TTM (24 h or 48 h), urine output (mL/kg/h) in the intensive care unit, event of shock (mean arterial pressure < 70 mmHg) after ROSC, daily serum creatinine level (mg/dL), RRT (yes or no), the type of RRT (continuous RRT or haemodialysis) and date of RRT (initiation, termination and duration), discharge date, survival status at discharge (yes or no) and Modified Rankin Scale (MRS) score at discharge. If the serum creatinine level was checked two or more times in a day, the highest value was recorded. All dates were calculated from the day of ROSC. The MRS score was used to assess neurological outcomes, with an MRS score of 0–3 being regarded as a good outcome and that of 4–6 as a poor outcome [12, 13].

### Statistical methods

Descriptive statistics are reported as medians (interquartile range) or means with standard deviation for continuous variables according to the normality of distribution. The normal distribution of data was analysed using the Shapiro–Wilk test or Kolmogorov–Smirnov test. Categorical variables are reported as frequency (percentage). Demographics and clinical differences between groups were assessed using Pearson’s chi-squared test or Fisher’s exact test, as appropriate. Comparisons of variables between groups were conducted using the two-sided Student *t* test or Mann–Whitney *U* test, as appropriate.

The association between predictors and outcome was quantified using odds ratio (OR) with 95% confidence interval (CI). To determine the independent factors associated with AKI development, recovery, and outcomes, we performed multivariate logistic regression analysis initially including all variables with *P* value < 0.2; we then applied a stepwise backward selection of the variables which remained significant. The Hosmer–Lemeshow test was used to evaluate the goodness of fit of the logistic regression model. The Cox regression analysis with time-varying covariate was used in the survival analysis to calculate the hazard ratio of AKI with respect to mortality. A *P* value < 0.05 was considered statistically significant. Statistical analyses were performed using IBM SPSS version 25.0 (IBM Corp., Armonk, NY, USA).

## Results

### Study population

**Fig. 1:**
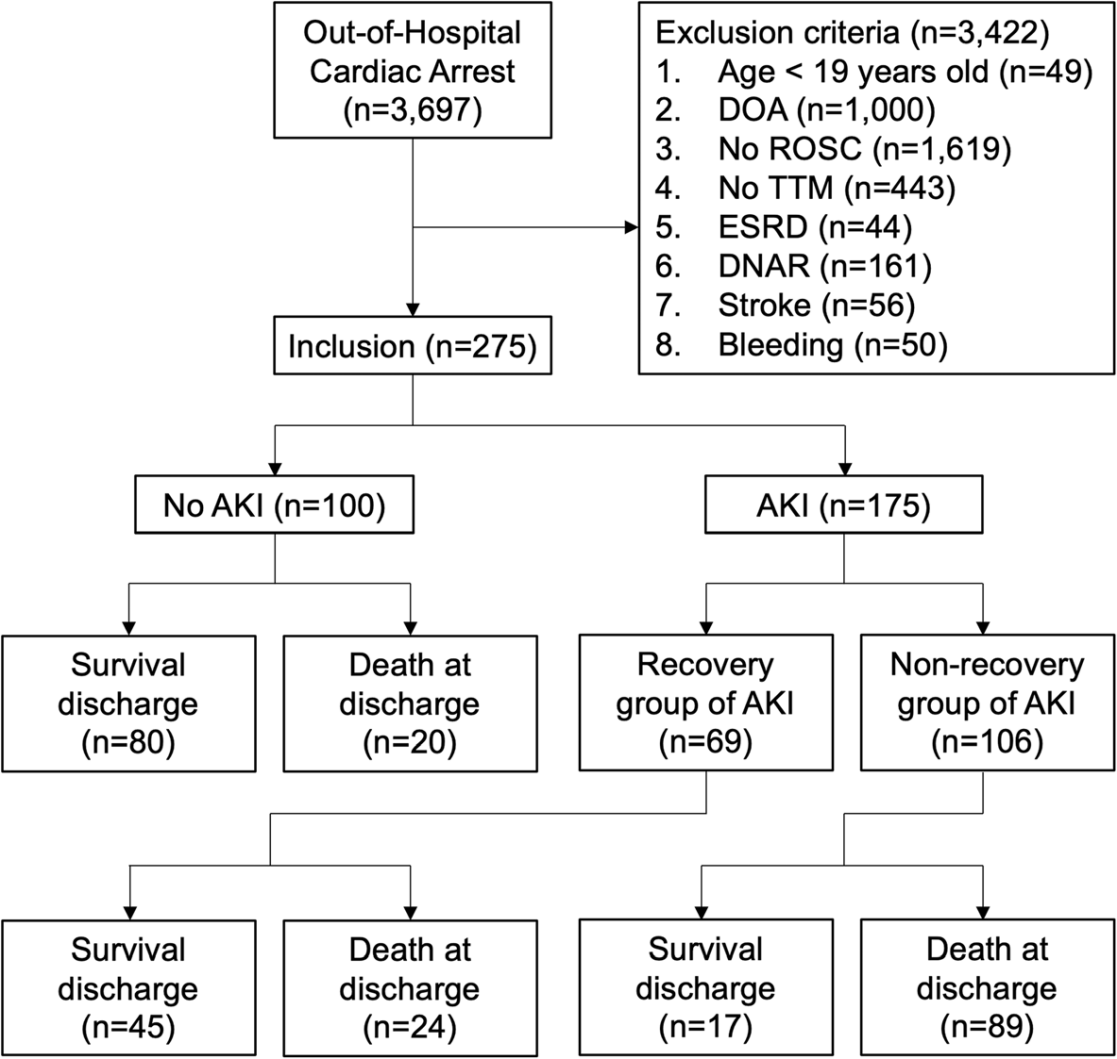
Flow chart of the study population. AKI, acute kidney injury; DNAR, do-not-attempt-resuscitation; DOA, death on arrival; ESRD, end-stage renal disease; ROSC, return of spontaneous circulation; TTM, targeted temperature management

Of the 3697 OHCA patients screened during the study period, 275 patients treated with TTM at six academic hospitals in South Korea were enrolled after excluding 3422 patients (Fig. 1). The baseline characteristics of the study population according to the development of and recovery from AKI are summarised in Tables 1 and 2.

**Table 1:** Baseline characteristics of the study population according to acute kidney injury development

Variable	All patients (*n* = 275)	No AKI (*n* = 100)	AKI (*n* = 175)	*P* value
Demographics				
Male sex	187/275 (68)	70/100 (70)	117/175 (67)	0.591
Age ≥ 60 years	139/275 (51)	40/100 (40)	99/175 (57)	*0.008*
Weight ≥ 60 kg	178/272 (65)	63/98 (64)	115/174 (66)	0.764
Medical history				
Heart failure	7/272 (3)	0/100 (0)	7/172 (4)	0.050
Hypertension	114/273 (42)	30/100 (30)	84/173 (49)	*0.003*
Diabetes mellitus	67/273 (25)	17/100 (17)	50/173 (29)	*0.028*
Chronic kidney disease	10/272 (4)	0/100 (0)	10/172 (6)	*0.015*
Resuscitation				
Arrest cause				0.841
Medical or uncertain	224/275 (82)	83/100 (83)	141/175 (81)	
Trauma	4/275 (2)	0/100 (0)	4/175 (2)	
Poisoning	3/275 (1)	1/100 (1)	2/175 (1)	
Drowning	1/275 (0)	1/100 (1)	0/175 (0)	
Asphyxia	43/275 (16)	15/100 (15)	28/175 (16)	
Arrest location				0.550
Home	134/275 (49)	47/100 (47)	87/175 (50)	
Workplace	10/275 (4)	3/100 (3)	7/175 (4)	
Sport	6/275 (2)	5/100 (5)	1/175 (1)	
Street	65/275 (24)	19/100 (19)	46/175 (26)	
Public building	56/275 (20)	24/100 (24)	32/175 (18)	
Nursing home	4/275 (1)	2/100 (2)	2/175 (1)	
Witnessed arrest	207/275 (75)	80/100 (80)	127/175 (73)	0.170
Bystander CPR	161/275 (59)	58/100 (58)	103/175 (59)	0.890
CPR time ≥ 20 min	135/242 (56)	34/86 (40)	101/156 (65)	*< 0.001*
Shockable rhythm	82/264 (31)	45/95 (47)	37/169 (22)	*< 0.001*
Adrenaline dose ≥ 4 mg	61/225 (27)	11/71 (16)	50/154 (33)	*0.008*
Post-resuscitation				
Shock	186/275 (68)	44/100 (44)	142/175 (81)	*< 0.001*
Coronary angiography	64/275 (23)	34/100 (34)	30/175 (17)	*0.001*
Target temperature = 33 °C	210/275 (76)	82/100 (82)	128/175 (73)	0.096
TTM duration = 24 h	224/275 (82)	84/100 (84)	140/175 (80)	0.412
Outcomes				
Survival discharge	142/275 (52)	80/100 (80)	62/175 (35)	*< 0.001*
MRS score 0 to 3 at discharge	72/275 (26)	47/100 (47)	25/175 (14)	*< 0.001*

*P* < 0.05 are presented in italics

*AKI* acute kidney injury, *CPR* cardiopulmonary resuscitation, *MRS* modified Rankin Scale, *RRT* renal replacement therapy, *TTM* targeted temperature management

**Table 2:** Baseline characteristics of the study population according to the recovery of acute kidney injury

Variable	All patients with AKI (*n* = 175)	AKI non-recovery group (*n* = 106)	AKI recovery group (*n* = 69)	*P* value
Characteristics of AKI				
AKI stage (initial)				*< 0.001*
Stage 1	120/175 (69)	57/106 (54)	63/69 (91)	
Stage 2	25/175 (14)	20/106 (19)	5/69 (7)	
Stage 3	30/175 (17)	29/106 (27)	1/69 (2)	
AKI stage (highest)				*< 0.001*
Stage 1	68/175 (39)	16/106 (15)	52/69 (75)	
Stage 2	30/175 (17)	20/106 (19)	10/69 (15)	
Stage 3	77/175 (44)	70/106 (66)	7/69 (10)	
Duration of AKI ≥ 4 days	88/175 (50)	66/106 (62)	22/69 (32)	*< 0.001*
RRT frequency	45/175 (26)	41/106 (39)	4/69 (6)	*< 0.001*
RRT duration ≥ 4 days	23/45 (51)	22/41 (54)	1/4 (25)	0.346
†RRT requirements at discharge	4/8 (50)	4/6 (67)	0/2 (0)	0.429
Demographics				
Male sex	117/175 (67)	70/106 (66)	47/69 (68)	0.775
Age ≥ 60 years	99/175 (57)	68/106 (64)	31/69 (45)	*0.012*
Weight ≥ 60 kg	115/174 (66)	71/105 (68)	44/69 (64)	0.600
Medical history				
Heart failure	7/172 (4)	6/104 (6)	1/68 (2)	0.247
Hypertension	84/173 (49)	56/105 (53)	28/68 (41)	0.118
Diabetes mellitus	50/173 (29)	37/105 (35)	13/68 (19)	*0.022*
Chronic kidney disease	10/172 (6)	9/104 (9)	1/68 (2)	0.091
Resuscitation				
Arrest cause				0.520
Medical or uncertain	141/175 (81)	88/106 (83)	53/69 (77)	
Trauma	4/175 (2)	2/106 (2)	2/69 (3)	
Poisoning	2/175 (1)	0/106 (0)	2/69 (3)	
Drowning	0/175 (0)	0/106 (0)	0/69 (0)	
Asphyxia	28/175 (16)	16/106 (15)	12/69 (17)	
Arrest location				0.680
Home	87/175 (50)	57/106 (54)	30/69 (44)	
Workplace	7/175 (4)	1/106 (1)	6/69 (9)	
Sport	1/175 (1)	0/106 (0)	1/69 (1)	
Street	46/175 (26)	27/106 (26)	19/69 (28)	
Public building	32/175 (18)	19/106 (18)	13/69 (19)	
Nursing home	2/175 (1)	2/106 (2)	0/69 (0)	
Witnessed arrest	127/175 (73)	76/106 (72)	51/69 (74)	0.748
Bystander CPR	103/175 (59)	61/106 (58)	42/69 (61)	0.662
CPR time ≥ 20 min	101/156 (65)	71/96 (74)	30/60 (50)	*0.002*
Shockable rhythm	37/169 (22)	19/101 (19)	18/68 (27)	0.238
Adrenaline dose ≥ 4 mg	50/154 (33)	40/96 (42)	10/58 (17)	*0.002*
Post-resuscitation				
Shock	142/175 (81)	96/106 (91)	46/69 (67)	*< 0.001*
Coronary angiography	30/175 (17)	13/106 (12)	17/69 (25)	*0.034*
Target temperature = 33 °C	128/175 (73)	76/106 (72)	52/69 (75)	0.593
TTM duration = 24 h	140/175 (80)	83/106 (78)	57/69 (83)	0.486
Outcomes				
Survival discharge	62/175 (35)	17/106 (16)	45/69 (65)	*< 0.001*
MRS score 0 to 3 at discharge	25/175 (14)	3/106 (3)	22/69 (32)	*< 0.001*

*P* < 0.05 are presented in italics

†Thirty-seven patients receiving renal replacement therapy (RRT) died before discharge. Therefore, whether or not RRT was required could only be assessed in eight patients

*AKI* acute kidney injury, *RRT* renal replacement therapy, *CPR* cardiopulmonary resuscitation, *TTM* targeted temperature management, *MRS* modified Rankin Scale

### Descriptive data

The median age of patients was 66 (55–73) years, and weight was 65 ± 12 kg, with most patients being male (187/275, 68%). The median time interval from call to arrival of emergency medical service was 7 (5–11) min. The median duration of CPR was 23 (12–32) min, and median dose of adrenaline (epinephrine) was 2 (1–4) mg.

### Clinical course of AKI

**Fig. 2:**
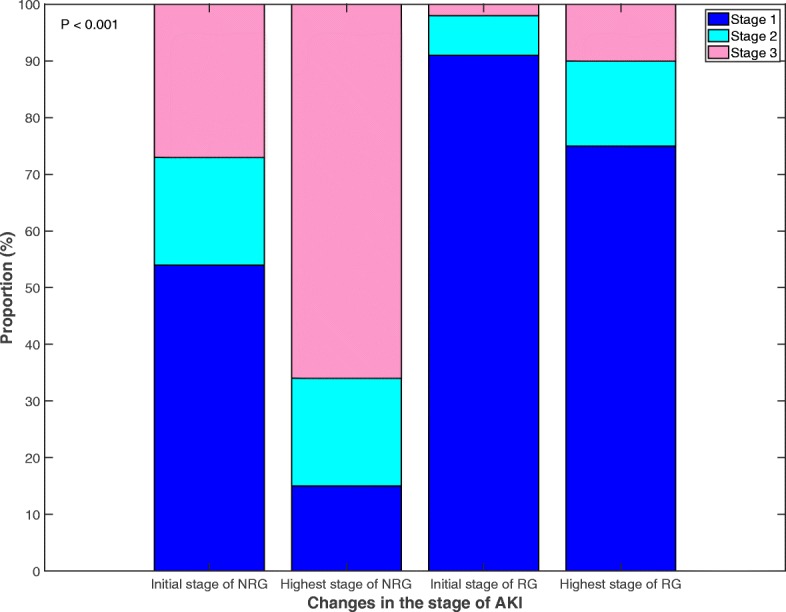
Changes in the stage of acute kidney injury after out-of-hospital cardiac arrest. AKI, acute kidney injury; NRG, non-recovery group; RG, recovery group

AKI developed in 175/275 (64%) patients and 69/175 (39%) patients recovered from AKI. In total, 169/175 (97%) patients were diagnosed with AKI based on serum creatinine level and 6/175 (3%) were diagnosed based on the urine output criteria. In most cases, AKI developed within three days of ROSC [155/175 (89%), median time to AKI development, 1 (1–2) day] and patients recovered within seven days of ROSC [59/69 (86%), median time to AKI recovery, 3 (2–7) days]. The median duration of AKI was 4 (2–7) days. Duration of AKI was significantly longer in the AKI non-recovery group than in the AKI recovery group [5 (2–9) vs. 1 (1–5) days; *P* < 0.001]. Most patients were diagnosed with AKI stage 1 initially [120/175 (69%)]. However, the number of stage 3 AKI patients increased from 30/175 (17%) to 77/175 (44%) after the initial diagnosis of AKI (Table 2). In addition, the proportions of patients at different AKI stages varied significantly between AKI non-recovery and recovery groups (Table 2 and Fig. 2; *P* < 0.001). RRT was conducted in 45/175 (26%) patients. The median duration of RRT was 4 (1–8) days. Use of RRT was significantly higher in the AKI non-recovery group than in the AKI recovery group [41/106 (39%) vs. 4/69 (6%); *P* < 0.001]. RRT requirements at discharge could only be evaluated in eight patients (4/8 [50%]), because 37 patients receiving RRT did not survive till discharge (Table 2). Four of these patients required RRT at discharge, two had fully recovered by discharge, and two were diagnosed with chronic kidney disease.

### Factors associated with AKI development and recovery

In all patients, hypertension and shock were potent predictors for the development of AKI (Table 3). Among patients with AKI, hypertension, CPR time ≥ 20 min, AKI stage 3 (highest), and shock were negatively associated with recovery from AKI (Table 3).

**Table 3:** Factors associated with acute kidney injury development and recovery in multivariate analysis

	Odds ratio	95% confidence interval	*P* value
AKI development			
Hypertension	3.011	1.434, 6.322	*0.004*
Shock	5.654	2.750, 11.626	*< 0.001*
AKI recovery			
Hypertension	0.357	0.137, 0.930	*0.035*
CPR time ≥ 20 min	0.275	0.104, 0.730	*0.010*
AKI stage 3 (highest)	0.130	0.040, 0.420	*0.001*
Shock	0.173	0.049, 0.605	*0.006*

*P* < 0.05 are presented in italics

*AKI* acute kidney injury, *CPR* cardiopulmonary resuscitation

AKI development: Hosmer and Lemeshow test: chi-square = 0.537; df = 4; *P* = 0.970

AKI recovery: Hosmer and Lemeshow test: chi-square = 8.980; df = 7; *P* = 0.254

### Impact of target temperature on the AKI

After adjusting for age, medical history, witnessed arrest, CPR time, shockable rhythm, epinephrine dose, shock, and coronary angiography, the target temperature was not associated with the development of AKI (the target temperature was eliminated in step 5 of a stepwise backward selection of variables; OR, 0.670; 95% CI, 0.280–1.601; *P* = 0.367). In addition, the target temperature was also not associated with recovery from AKI (OR, 1.207; 95% CI, 0.605–2.411; *P* = 0.593).

### Impact of development of and recovery from AKI on outcomes

Survival discharge and good neurological outcome at discharge (MRS scores, 0–3) were observed in a significantly higher number of non-AKI patients than in AKI patients [survival discharge 80/100 (80%) vs. 62/175 (35%); *P* < 0.001; good neurological outcome, 47/100 (47%) vs. 25/175 (14%); *P* < 0.001, Table 1]. In addition, the proportion of patients with survival discharge and good neurological outcome at discharge was significantly higher in the AKI recovery group than in the AKI non-recovery group [survival discharge, 45/69 (65%) vs. 17/106 (16%); *P* < 0.001; good neurological outcome 22/69 (32%) vs. 3/106 (3%); *P* < 0.001].

In multivariate analysis, the development of AKI was negatively associated with survival discharge (adjusted OR, 0.238; 95% CI, 0.102–0.555; *P* = 0.001; Table 4). In patients with AKI, recovery from AKI was a potent predictor of survival discharge (adjusted OR, 8.308; 95% CI, 3.120–22.123; *P* < 0.001; Table 4). Development of AKI was not associated with good neurological outcome at discharge; however, recovery from AKI was a potent predictor of good neurological outcome at discharge (adjusted OR, 36.822; 95% CI 4.097–330.926; *P* = 0.001; Table 5).

**Table 4:** Factors associated with survival discharge in multivariate analysis

	Odds ratio	95% confidence interval	*P* value
All patients			
Adrenaline dose ≥ 4 mg	0.428	0.187, 0.978	*0.044*
Shock	0.089	0.034, 0.232	*< 0.001*
Coronary angiography	10.955	3.412, 35.172	*< 0.001*
AKI	0.238	0.102, 0.555	*0.001*
AKI patients			
Shock	0.178	0.051, 0.626	*0.007*
Coronary angiography	12.084	3.116, 46.856	*< 0.001*
AKI recovery	8.308	3.120, 22.123	*< 0.001*

*P* < 0.05 are presented in italics

*AKI* acute kidney injury

All patients: Hosmer and Lemeshow test: chi-square = 8.954; df = 7; *P* = 0.256

AKI patients: Hosmer and Lemeshow test: chi-square = 5.315; df = 4; *P* = 0.256

**Table 5:** Factors associated with good neurological outcome in multivariate analysis

	Odds ratio	95% confidence interval	*P* value
All patients			
Shockable rhythm	3.085	1.057, 9.006	*0.039*
CPR time ≥ 20 min	0.235	0.081, 0.679	*0.007*
Shock	0.160	0.055, 0.465	*0.001*
Coronary angiography	14.087	4.291, 46.248	*< 0.001*
AKI patients			
Coronary angiography	12.644	2.712, 58.952	*0.001*
AKI recovery	36.822	4.097, 330.926	*0.001*

*P* < 0.05 are presented in italics

*AKI* acute kidney injury, CPR cardiopulmonary resuscitation

All patients: Hosmer and Lemeshow test: chi-square = 5.207; df = 6; *P* = 0.518

AKI patients: Hosmer and Lemeshow test: chi-square = 0.733; df = 2; *P* = 0.693

### Subgroup analysis in patients with good neurological outcomes

There were no significant differences in the distribution of MRS scores according to AKI development, recovery, and RRT in the cohort with good neurological outcomes at discharge (see Additional file 1, Tables S1 to S3). Only two patients (MRS 2 and 3) were treated with RRT (Additional file 1: Table S3), one of whom (MRS 3) still required RRT at discharge.

### Recovery rate after AKI according to the stages of AKI

Recovery rate after AKI was significantly higher in patients with stage 1 AKI than in those with stage 2 or 3 AKI [initial stage: stage 1, 63/120 (53%); stage 2, 5/25 (20%); stage 3, 1/30 (3%); *P* < 0.001 and highest stage: stage 1, 52/68 (77%); stage 2, 10/30 (33%); stage 3, 7/77 (9%); *P* < 0.001).

### Survival analysis

Cox regression analysis with time-varying covariate of AKI showed that patients with AKI had a higher risk of death than those without AKI (hazard ratio, 2.813; 95% confidence interval, 1.628–4.859; *P* < 0.001; Table 6).

**Table 6:** Cox regression analysis with time-varying covariate of development of acute kidney injury showing the effect of five variables on the risk of death

Variable	Coefficient	Standard Error	Wald	*P* value	Hazards ratio	95% CI
Witnessed arrest	−0.485	0.218	4.968	*0.026*	0.615	0.402, 0.943
Adrenaline dose ≥ 4 mg	0.611	0.211	8.419	*0.004*	1.843	1.219, 2.784
Coronary angiography	−1.629	0.468	12.138	*< 0.001*	0.196	0.078, 0.490
Shock	1.446	0.374	14.976	*< 0.001*	4.244	2.041, 8.826
†T_COV_AKI	1.034	0.279	13.746	*< 0.001*	2.813	1.628, 4.859

*P* < 0.05 are presented in italics

†T_COV_AKI means time-varying covariate of AKI development

*AKI* acute kidney injury, *CI* confidence interval

## Discussion

To the best of our knowledge, the present study is the first to investigate the recovery rate after AKI and the association between the recovery from AKI and outcomes after OHCA in patients treated with TTM. Although the natural course of AKI and the relationship between AKI and clinical outcomes in other critical care areas such as sepsis have been previously investigated, the clinical outcomes in post-cardiac arrest care are still uncertain [14].

The overall incidence of AKI in our cohort (64%) was slightly higher than the weighted mean prevalence of AKI (52%; range, 37–81%) in a previous systematic review because the proportion of patients with a non-shockable rhythm in our cohort was high (69%) [3]. In South Korea, non-shockable rhythms occupy a large proportion of initial rhythms [15]. Because shockable rhythms are associated with a significantly lower risk of developing AKI, the incidence of AKI in our cohort might be higher than that in previous reports [4, 6]. The same reason may have affected the low recovery rate after AKI in our cohort (39%); however, the proportions of shockable rhythms were not significantly different between the AKI non-recovery and recovery groups [19/101 (19%) vs. 18/68 (27%); *P* = 0.238]. A previous study reported that the renal function returned to normal before hospital discharge in 67/83 (81%) survivors [3]. In our results, the recovery rate increased up to 73% when we considered only the patients with survival discharge (45/62 patients). However, we could not determine whether the recovery rate after AKI would be different depending on the proportion of shockable rhythms. Thus, further studies including higher proportion of shockable rhythms are needed.

In contrast to the negative association between the development of AKI and survival discharge, there was a strong positive association between recovery from AKI and outcomes (acceptance of our hypothesis). This association may be caused by changes in the AKI stages after the initial diagnosis of AKI because an increase in the proportion of patients with stage 3 AKI led to an increase in mortality in this study. In the case of the AKI non-recovery group, the proportion of patients with stage 3 AKI increased from 27 to 66% (Fig. 2).

The association between the development of AKI and neurological outcome had been reported several times [1, 4]. Although the development of AKI was significantly associated with survival at discharge, it was not associated with good neurological outcome at discharge. This might be attributed to small number of participants. However, multivariate analysis showed that recovery from AKI was a potent predictor of survival and good neurological outcome at discharge. Naturally, we should interpret these results carefully because the characteristics of the OHCA patients are significantly different from critical care patients, such as those with severe sepsis [14, 16]. Whole body ischaemia and reperfusion leads to post-cardiac arrest syndrome, including brain injury, myocardial dysfunction, systemic ischaemia, and reperfusion response [16]. Various factors, including the duration of ischaemia, cause of cardiac arrest, OHCA interventions, and the patient’s baseline health status, could affect neurological outcomes in OHCA patients. Therefore, to determine whether the development of and recovery from AKI could affect the long-term neurological and functional outcomes of OHCA patients, further studies are warranted. In addition, patients’ death before recovery of AKI could exaggerate the outcome of the AKI recovery group compared to that of the AKI non-recovery group. In addition, patients’ death before the recovery of AKI might be caused by reasons (competing risk problems) other than AKI [17]. To decrease competing risk problems, we conducted subgroup analysis after excluding patients who died within 48 h since ROSC (Additional file 1: Table S4). Development of and recovery from AKI were significant predictors of survival discharge in the multivariate analysis even after excluding patients who died within 48 h (Additional file 1: Table S5). In addition, recovery from AKI was a potent predictor of good neurological outcome at discharge in patients with AKI (Additional file 1: Table S6). Most of the results of the multivariate analysis did not change after excluding patients who died within 48 h since ROSC.

Neither the incidence of AKI nor recovery of AKI was significantly influenced by target temperature (33 °C vs. 36 °C) in our cohort. However, the exact effect of target temperatures on the development of AKI could not be determined in this study because the target temperature was not randomised in our cohort. Recently, the post hoc analysis of TTM trial (randomised clinical trial for comparing the TTM 33 °C versus 36 °C after OHCA) [10] for evaluating the effect of target temperature on the development of AKI has been reported [18, 19]. Although the incidence of AKI was higher in the TTM-33 group compared to that in the TTM-36 group (49% versus 40%), there was no association between the target temperature and development of AKI based on the logistic regression analysis [18]. We could not determine whether there was a difference in the recovery rate of AKI according to the target temperature because the recovery rate according to the target temperature had not been reported in that study.

The appropriate time to initiate RRT in a critical care setting has not been defined [20]. Studies on the issues of early versus delayed initiation of RRT show heterogeneous results in a critical care setting [21–23]. In the case of OHCA treated with TTM, studies on early initiation of RRT have not yet been conducted. In our cohort, RRT was mostly conducted after the patient reached Stage 3 AKI (95%). Therefore, we could not verify the preventive effect of RRT in our study. Another study including a large cohort is warranted to verify the effect of RRT on the outcomes of the OHCA treated with TTM.

### Limitations

The present study has several limitations. First, there was a higher risk of selection bias because the data of the present study were collected retrospectively. A prospective study will be needed to confirm our findings. Second, although the present study was conducted using a multicentre cohort, the size of the cohort was relatively small. In addition, all study sites were located in South Korea. Therefore, our results cannot be representatives of all OHCA patients. Third, determining the development of and recovery from AKI based on serum creatinine level could lead to underestimation of the development of AKI or overestimation of recovery from AKI compared to an approach based on the estimated glomerular filtration rate calculated from cystatin C [24]. In addition, decreased muscle metabolism in critically ill patients could also lead to an overestimation of renal function [25]. Therefore, the incidence and recovery rates of AKI in our cohort might be affected by the use of serum creatinine levels to diagnose AKI in most patients. Fourth, we could not determine the effect of RRT in the present study because most of the patients (80%) treated with RRT did not survive to discharge. Further studies are needed to evaluate the efficacy of RRT in OHCA patients treated with TTM. Fifth, although we determined the development of AKI by considering both serum creatinine level and hourly urine output, we did not record the data on hourly urine output. Therefore, serial changes in hourly urine output could not be presented in the results. Sixth, only a small number of patients were included from the screened OHCA patients (275/3,697 [7%]). In South Korea, the emergency medical service providers do not have the legal right to declare death. All patients with OHCA are transported to the emergency to confirm death, with or without CPR, even if they show obvious signs of death (e.g. postmortem lividity and rigour mortis). Therefore, 71% (2619/3697) of the screened patients were initially excluded from our cohort. The important exclusion was the absence of treatment with TTM (443/3697 patients [12%]). There were several reasons for this exclusion. Most of the institutions in South Korea use surface cooling or intravascular cooling devices for strict temperature control, including rewarming speed. However, the disposable components of these devices are not covered by the South Korean national health insurance system. Approximately 1500 to 3500 USD is needed for TTM treatment, in addition to the basic costs of post-cardiac arrest care and intensive care. Therefore, some of the patients’ legal surrogates did not agree to TTM. In addition to the financial issue, TTM itself has several contraindications. For example, refractory shock that does not respond to fluid or vasopressors, severe sepsis, poor neurological condition before cardiac arrest, pregnancy, arrhythmia that does not respond to treatment or coronary vasospasm, active bleeding or thrombolytic use, terminal illness before cardiac arrest, DNAR orders, and comatose patients with other aetiologies. The above reasons might have limited patient inclusion in the present study.

## Conclusions

In our cohort of adult OHCA patients treated with TTM (*n* = 275), the recovery rate from AKI after OHCA was 39%, and recovery from AKI was a potent predictor of survival and good neurological outcome at discharge.

## Additional file

Additional file 1: **Table S1.** The distribution of modified Rankin Scale scores according to the development of acute kidney injury in patients with good neurological outcomes at discharge. **Table S2.** The distribution of modified Rankin Scale scores according to the recovery from acute kidney injury in patients with good neurological outcomes at discharge. **Table S3.** The distribution of modified Rankin Scale scores according to renal replacement therapy in patients with good neurological outcomes at discharge. **Table S4.** Baseline characteristics of the study population according to the recovery of acute kidney injury after excluding patients who died within 48 h since return of spontaneous circulation. **Table S5.** Factors associated with survival discharge in multivariate analysis after excluding patients who died within 48 h since return of spontaneous circulation. **Table S6.** Factors associated with good neurological outcome in multivariate analysis after excluding patients who died within 48 h since return of spontaneous circulation. (DOCX 22 kb)

## Data Availability

The datasets used and/or analysed during the current study are available from the corresponding author on reasonable request.

## Abbreviations

AKI: Acute kidney injury
CI: Confidence interval
CPR: Cardiopulmonary resuscitation
DNAR: Do-not-attempt resuscitation
ESRD: End-stage renal disease
KDIGO: Kidney Disease: Improving Global Outcomes
MRS: Modified Rankin scale
OHCA: Out-of-hospital cardiac arrest
OR: Odds ratio
ROSC: Return of spontaneous circulation
RRT: Renal replacement therapy
TTM: Targeted temperature management
