# Supplementary material for: Recovery from acute kidney injury as a potent predictor of survival and good neurological outcome at discharge after out-of-hospital cardiac arrest
Source: Crit Care. 2019 Jul 15;23:256. doi: 10.1186/s13054-019-2535-1 (PMC6632185; doi:10.1186/s13054-019-2535-1)
Supplement: Supplementary file 1 — Table S1. The distribution of modified Rankin Scale scores according to the development of acute kidney injury in patients with good neurological outcomes at discharge. Table S2. The distribution of modified Rankin Scale scores according to the recovery from acute kidney injury in patients with good neurological outcomes at discharge. Table S3. The distribution of modified Rankin Scale scores according to renal replacement therapy in patients with good neurological outcomes at discharge. Table S4. Baseline characteristics of the study population according to the recovery of acute kidney injury after excluding patients who died within 48 h since return of spontaneous circulation. Table S5. Factors associated with survival discharge in multivariate analysis after excluding patients who died within 48 h since return of spontaneous circulation. Table S6. Factors associated with good neurological outcome in multivariate analysis after excluding patients who died within 48 h since return of spontaneous circulation. (DOCX 22 kb) [file 13054_2019_2535_MOESM1_ESM.docx]

**Supplementary materials**

**Definition of acute kidney injury (AKI) in the Kidney Disease: Improving Global Outcomes (KDIGO) guidelines**

As defined in the KDIGO guidelines, Stage 1 AKI refers to an absolute increase in serum creatinine level by ≥0.3 mg/dL or a 1.5–1.9-fold increase from the baseline value or a urine output level of <0.5 mL/kg/h for 6–12 h; Stage 2 AKI refers to a 2.0–2.9-fold increase in serum creatinine level from the baseline value or a urine output level of <0.5 mL/kg/h for ≥12 h and Stage 3 AKI refers to a 3.0-fold increase in serum creatinine level from the baseline value, an increase in serum creatinine level by ≥4.0 mg/dL, initiation of renal replacement therapy, a urine output level of <0.3 mL/kg/h for ≥24 h, or anuria for ≥12 h.

Table S1. The distribution of modified Rankin Scale scores according to the development of acute kidney injury in patients with good neurological outcomes at discharge

|  | Total (n = 72) | No AKI (n = 47) | AKI (n = 25) |
| --- | --- | --- | --- |
| MRS 0 | 1/72 (1) | 1/47 (2) | 0/25 (0) |
| MRS 1 | 21/72 (29) | 15/47 (32) | 6/25 (24) |
| MRS 2 | 33/72 (46) | 20/47 (43) | 13/25 (52) |
| MRS 3 | 17/72 (24) | 11/47 (23) | 6/25 (24) |

*P* = 0.500

AKI, acute kidney injury; MRS, modified Rankin Scale

Table S2. The distribution of modified Rankin Scale scores according to the recovery from acute kidney injury in patients with good neurological outcomes at discharge

|  | Total (n = 25) | No recovery of AKI (n = 3) | Recovery of AKI (n = 22) |
| --- | --- | --- | --- |
| MRS 0 | 0/25 (0) | 0/3 (0) | 0/22 (0) |
| MRS 1 | 6/25 (24) | 0/3 (0) | 6/22 (27) |
| MRS 2 | 13/25 (52) | 2/3 (67) | 11/22 (50) |
| MRS 3 | 6/25 (24) | 1/3 (33) | 5/22 (23) |

*P* = 0.384

AKI, acute kidney injury; MRS, modified Rankin Scale

Table S3. The distribution of modified Rankin Scale scores according to renal replacement therapy in patients with good neurological outcomes at discharge

|  | Total (n = 72) | No RRT (n = 70) | RRT (n = 2) |
| --- | --- | --- | --- |
| MRS 0 | 1/72 (1) | 1/70 (1) | 0/2 (0) |
| MRS 1 | 21/72 (29) | 21/70 (30) | 0/2 (0) |
| MRS 2 | 33/72 (46) | 32/70 (46) | 1/2 (50) |
| MRS 3 | 17/72 (24) | 16/70 (23) | 1/2 (50) |

*P* = 0.274

MRS, modified Rankin Scale; RRT, renal replacement therapy

Table S4. Baseline characteristics of the study population according to the recovery of acute kidney injury after excluding patients who died within 48 hours since return of spontaneous circulation

| Variable | All patients with AKI  (n = 157) | AKI non-recovery group  (n = 88) | AKI recovery group  (n = 69) | *P*-value |
| --- | --- | --- | --- | --- |
| **Characteristics of AKI** | | | | |
| AKI stage (initial) |  |  |  | **<0.001** |
| Stage 1 | 108/157 (69) | 45/88 (51) | 63/69 (91) |  |
| Stage 2 | 22/157 (14) | 17/88 (19) | 5/69 (7) |  |
| Stage 3 | 27/157 (17) | 26/88 (30) | 1/69 (2) |  |
| AKI stage (highest) |  |  |  | **<0.001** |
| Stage 1 | 62/157 (40) | 10/88 (11) | 52/69 (75) |  |
| Stage 2 | 26/157 (16) | 16/88 (18) | 10/69 (15) |  |
| Stage 3 | 69/157 (44) | 62/88 (71) | 7/69 (10) |  |
| Duration of AKI ≥ 4 days | 88/157 (56) | 66/88 (75) | 22/69 (32) | **<0.001** |
| RRT frequency | 40/157 (26) | 36/88 (41) | 4/69 (6) | **<0.001** |
| RRT duration ≥ 4 days | 23/40 (58) | 22/36 (61) | 1/4 (25) | 0.294 |
| RRT requirements at discharge | 4/8 (50) | 4/6 (67) | 0/2 (0) | 0.429 |
| **Demographics** | | | | |
| Male sex | 105/157 (67) | 58/88 (66) | 47/69 (68) | 0.771 |
| Age ≥ 60 years | 85/157 (54) | 54/88 (61) | 31/69 (45) | **0.040** |
| Weight ≥ 60 kg | 103/156 (66) | 59/87 (68) | 44/69 (64) | 0.596 |
| **Medical history** | | | | |
| Heart failure | 7/154 (5) | 6/88 (7) | 1/68 (2) | 0.134 |
| Hypertension | 74/155 (48) | 46/87 (53) | 28/68 (41) | 0.148 |
| Diabetes mellitus | 44/155 (28) | 31/87 (36) | 13/68 (19) | **0.024** |
| Chronic kidney disease | 10/154 (7) | 9/86 (11) | 1/68 (2) | **0.043** |
| **Resuscitation** | | | | |
| Arrest cause |  |  |  | 0.788 |
| Medical or uncertain | 124/157 (79) | 71/88 (81) | 53/69 (77) |  |
| Trauma | 4/157 (3) | 2/88 (2) | 2/69 (3) |  |
| Poisoning | 2/157 (1) | 0/88 (0) | 2/69 (3) |  |
| Drowning | 0/157 (0) | 0/88 (0) | 0/69 (0) |  |
| Asphyxia | 27/157 (17) | 15/88 (17) | 12/69 (17) |  |
| Arrest location |  |  |  | 0.770 |
| Home | 77/157 (49) | 47/88 (53) | 30/69 (44) |  |
| Workplace | 7/157 (5) | 1/88 (1) | 6/69 (9) |  |
| Sport | 1/157 (1) | 0/88 (0) | 1/69 (1) |  |
| Street | 40/157 (26) | 21/88 (24) | 19/69 (28) |  |
| Public building | 30/157 (19) | 17/88 (19) | 13/69 (19) |  |
| Nursing home | 2/157 (1) | 2/88 (2) | 0/69 (0) |  |
| Witnessed arrest | 113/157 (72) | 62/88 (71) | 51/69 (74) | 0.632 |
| Bystander CPR | 95/157 (61) | 53/88 (60) | 42/69 (61) | 0.935 |
| CPR time ≥ 20 min | 90/138 (65) | 60/78 (77) | 30/60 (50) | **0.001** |
| Shockable rhythm | 36/152 (24) | 18/84 (21) | 18/68 (27) | 0.467 |
| Adrenaline dose ≥ 4 mg | 44/137 (32) | 34/79 (43) | 10/58 (17) | **0.001** |
| **Post-resuscitation** | | | | |
| Shock | 124/157 (79) | 78/88 (89) | 46/69 (67) | **0.001** |
| Coronary angiography | 29/157 (19) | 12/88 (14) | 17/69 (25) | 0.078 |
| Target temperature = 33°C | 118/157 (75) | 66/88 (75) | 52/69 (75) | 0.958 |
| TTM duration = 24 h | 123/157 (78) | 66/88 (75) | 57/69 (83) | 0.251 |
| **Outcomes** | | | | |
| Survival discharge | 62/157 (40) | 17/88 (19) | 45/69 (65) | **<0.001** |
| MRS score 0 to 3 at discharge | 25/157 (16) | 3/88 (3) | 22/69 (32) | **<0.001** |

*P* < 0.05 are presented in bold.

AKI, acute kidney injury; RRT, renal replacement therapy; CPR, cardiopulmonary resuscitation; TTM, targeted temperature management; MRS, modified Rankin Scale

Table S5. Factors associated with survival discharge in multivariate analysis after excluding patients who died within 48 hours since return of spontaneous circulation

|  | Odds ratio | 95% confidence interval | *P*-value |
| --- | --- | --- | --- |
| **All patients** | | | |
| Shock | 0.095 | 0.035, 0.257 | **<0.001** |
| Coronary angiography | 11.581 | 3.296, 40.684 | **<0.001** |
| AKI | 0.259 | 0.107, 0.628 | **0.003** |
| **AKI patients** | | | |
| Bystander CPR | 2.796 | 1.049, 7.456 | **0.040** |
| Shock | 0.196 | 0.056, 0.680 | **0.010** |
| Coronary angiography | 12.963 | 3.084, 54.482 | **<0.001** |
| AKI recovery | 6.694 | 2.485, 18.032 | **<0.001** |

*P* < 0.05 are presented in bold.

AKI, acute kidney injury; CPR, cardiopulmonary resuscitation

All patients: Hosmer and Lemeshow Test: Chi-square = 6.999; df = 8; *P* = 0.537

AKI patients: Hosmer and Lemeshow Test: Chi-square = 5.373; df = 5; *P* = 0.372

Table S6. Factors associated with good neurological outcome in multivariate analysis after excluding patients who died within 48 hours since return of spontaneous circulation

|  | Odds ratio | 95% confidence interval | *P*-value |
| --- | --- | --- | --- |
| **All patients** | | | |
| Shockable rhythm | 3.224 | 1.102, 9.432 | **0.033** |
| CPR time ≥ 20 min | 0.206 | 0.069, 0.617 | **0.005** |
| Coronary angiography | 15.134 | 4.513, 50.754 | **<0.001** |
| Shock | 0.189 | 0.064, 0.557 | **0.003** |
| **AKI patients** | | | |
| Coronary angiography | 12.437 | 2.667, 57.994 | **0.001** |
| AKI recovery | 31.243 | 3.424, 285.079 | **0.002** |

*P* < 0.05 are presented in bold.

AKI, acute kidney injury; CPR, cardiopulmonary resuscitation

All patients: Hosmer and Lemeshow Test: Chi-square = 4.101; df = 7; *P* = 0.768

AKI patients: Hosmer and Lemeshow Test: Chi-square = 0.663; df = 2; *P* = 0.718
